# Changes in Reported Symptoms Attributed to Office Environments in Sweden between 1995 and 2020

**DOI:** 10.3390/ijerph191811434

**Published:** 2022-09-11

**Authors:** Della Egfors, Anita Gidlöf Gunnarsson, Niklas Ricklund

**Affiliations:** 1 Department of Occupational and Environmental Medicine, Faculty of Medicine and Health, School of Medical Sciences, Örebro University, SE 70182 Orebro, Sweden; 2 Department of Occupational and Environmental Health, Faculty of Business, Science and Engineering, Örebro University, SE 70182 Orebro, Sweden

**Keywords:** non-specific building-related symptoms, sick building syndrome, office worker, indoor work environment, occupational medicine

## Abstract

Non-specific building-related symptoms (NBRSs) describe various symptoms in those affected. Questionnaires are the first step in investigating suspected NBRSs in office environments and have been used for over two decades. However, changes in reporting of symptoms among office workers over time are currently unknown. The overall aim was thus to investigate if reported symptoms and perceived causality to the office environment have changed during 25 years of using the MM 040 NA Office questionnaire. A cross-sectional study of 26,477 questionnaires from 1995–2020 was conducted, where 12 symptoms and perceived causality to office environment were examined using logistic regression analyses of 5-year groups adjusted for sex and atopy. Reporting trends in the year groups varied slightly among symptoms, but eight symptoms were statistically significant in the 2015–2020 group compared to the 1995–1999 group. Seven symptoms had increased: fatigue, heavy-feeling head, headache, difficulties concentrating, itchy/irritated eyes, congested/runny nose, and dry/red hands. One symptom decreased: hoarseness/dry throat. Perceived causality of symptoms to the office environment decreased to a statistically significant degree in 2015–2020 for 11 symptoms, and there was an overall trend of decreasing perceived causality throughout the year groups for most symptoms. The observed time trends suggest a need for up-to-date reference data, to keep up with changes in symptom reporting in office environments over time.

## 1. Introduction

Most people spend the majority of their time indoors, which places demands on interior environments to facilitate health and wellbeing [1,2,3]. Indoor work environments such as offices can affect physical and mental health [4]. The impact has been known about for two centuries, and several factors have been shown to increase the risk of a range of symptoms and decrease productivity in office workers [5,6,7]. Symptoms related to a specific building can be divided into two groups: building-related illness (BRI) and non-specific building-related symptoms (NBRSs).

BRI includes diseases, such as Legionnaires’ disease, where air contaminations or other clear environmental problems cause a specific pathological condition in the exposed individuals, and objective findings support the diagnosis [5,8,9]. NBRSs describe a variety of symptoms that are common in the general population. Symptoms include mucosal symptoms such as eye irritation, runny or congested nose, cough, and dry throat, as well as skin-related symptoms such as dry, flushed, or itching skin, and general symptoms such as fatigue, headache, and concentrating difficulties. In NBRSs, symptoms typically increase with exposure to the building and mitigate after leaving [4,10]. The phenomenon of these symptoms with perceived causality to a building, called the “sick building syndrome”, was first reported in the 1960s; reporting increased in the 1970s and 1980s [11]. The term has thereafter been questioned due to its unclear definition, and NBRSs has become a more widespread and accepted term [6,9]. The definitions of NBRSs and BRI differ regarding the variation of symptoms presented in NBRSs as well as the noticeable time relationship and the few objective findings in NBRSs.

Personal factors such as atopy and female sex have been previously shown to also be important risk factors for NBRSs [7,8,12]. Atopy is a hereditary tendency to develop antibodies to common environmental antigens, with a consequently higher risk of developing diseases such as atopic eczema, allergic rhinitis (hay fever), and allergic asthma [13]. Previous studies have suggested allergic asthma and allergic rhinitis as risk factors for NBRS [6,14]. One study discussed crossover mechanisms between immunological inflammation and neurogenic inflammation as a possible contributor to the strong associations between NBRSs and allergy [6]. People with asthma or allergies are often more sensitive to moist or insufficient ventilation, with worsening of symptoms [9,14]. Women are more often than men affected by NBRSs [7,8,9,15]. Suggestions of causes of the difference have been differences in work tasks or office environmental exposures [8], or different perceptions of psychosocial work conditions between sexes and different reactions to work-related stress [12].

Psychosocial stress in the work environment is of significance for symptom reporting. Work-related stress was associated with more symptoms in previous studies [12,15]. A relationship between psychosocial stress and NBRSs through neural cross-sensitization and stress-related inflammation has been suggested [6]. Stress can also negatively affect atopic eczema and is believed to increase sensitivity to office environmental factors [9].

Exposure to adverse environmental factors in the office environment, such as air pollutants, insufficient ventilation, uncomfortable temperatures, poor air quality, excessive noise, harsh lighting, vibration, or psychosocial stress, can affect perception of the office environment and reporting of symptoms [3,9,16,17]. Formaldehyde, for example, in office furniture, is a known irritant of eyes and airways [17]. Contact with chemical substances such as plastic and rubber chemicals, solvents, perfume, and metals can cause allergic or irritant contact eczema [14,18]. Noise can cause general symptoms such as fatigue, difficulties concentrating, and headache. In the office landscape, this can come from overhearing conversations and from office equipment [9]. Noise can also negatively affect performance and increase stress [14]. Exposure to dampness has been shown to exacerbate existing asthma and is also suggested to induce adult-onset asthma [19]. Previous experience of symptoms attributed to indoor environments can result in continued increased sensitivity to new exposures in another building [9]. On an individual level, causality between exposure and symptoms in indoor environments is seldom evident. Complexity is driven by the many exposure factors and differing symptom responses [2,20].

Assessment strategies typically involve systematic investigations of the building, involving technical aspects and questionnaires about environmental perceptions and perceived symptoms among workers—including temporal symptom patterns—to rule out other medical causes. Investigation methodologies utilized in Sweden are, e.g., the SWESIAQ methodology and the Örebro model [9,21]. The Örebro model for systematic investigation of indoor environments was developed in the 1980s, based on strategies from the WHO. The purpose is to quantify subjective experiences and range of symptoms. The results are evaluated by comparison to reference environments. The Örebro model includes questionnaires developed for different kinds of indoor environments, e.g., offices, schools, preschools, and hospitals. The Örebro questionnaires have been used nationally in indoor environment investigations in Sweden for more than two decades [21].

It can reasonably be assumed that patterns of questionnaire responses have been affected by changes over time in the office environment. For example, methods for construction of buildings, office layouts, climate comfort systems, emissions and chemicals emitted from available materials, and choices of furnishing all contribute to indoor air quality, as do other factors, such as noise and psychosocial contexts. The objective of this study was to investigate if reported symptoms and attribution to the office environment from the Örebro model questionnaire have changed over time between 1995 and 2020 in Sweden.

## 2. Materials and Methods

### 2.1. Study Design

This was a cross-sectional study of “MM 040 NA Office” questionnaires, collected by the Department of Occupational and Environmental Medicine in Sweden between the years 1995 and 2020. The questionnaires were completed by Swedish office workers in cases where symptoms attributed to the office environment were suspected among the employees, which was why the workplace had initiated contact with the Department of Occupational and Environmental Medicine and requested a survey. The questionnaire answers for the study were retrieved from a database at the Department in Örebro, which included questionnaires collected nationwide from year 1995 to 2014, and thereafter from four out of 21 counties (Örebro, Södermanland, Värmland, and Västmanland) from the years 2015 to 2020.

### 2.2. Exclusion Criteria

The initial dataset consisted of 26,478 questionnaires completed between 1995 and 30 April 2020. Exclusion criteria were age below 18 or over 70, excluding one questionnaire where the person was under 18. The final study material consisted of 26,477 questionnaires originating from 432 Swedish office workplaces.

### 2.3. The MM 040 NA Questionnaire

The questionnaire was administered in Swedish and contained items about the respondents’ background (e.g., sex, age, smoking habits), working conditions (e.g., working hours), perceived office environment (e.g., temperature, draft, dry air, smell), psychosocial working conditions, and symptoms (e.g., fatigue, headache, eye and nose irritation, skin-related) [22]. The question “Have you or have you ever had eczema, hay fever or asthma” was chosen as indicator for atopy. Questions about associated diseases such as allergic rhinitis and asthma have previously been shown to be good indicators of atopy in questionnaires [23]. Symptoms were evaluated by two questions: (i) regarding how often each symptom was experienced during a three-month recall period (“Yes often” meaning at least once every week, “Yes sometimes”, “Never”); and (ii) if they answered “Yes often”, they were requested to answer a follow-up question about if the workplace caused the symptom (“Yes”, “No”, “Don’t know”).

### 2.4. Statistics

Questionnaires were subdivided into five 5-year groups based on year of filling in the questionnaire: 1995–1999, 2000–2004, 2005–2009, 2010–2014, and 2015–2020. The year group 1995–1999 was selected as reference group for the analyses. Logistic regression analysis adjusted for sex and atopy status was used to study associations of questionnaire answers between the reference year group and later year groups. Answers regarding symptoms and perceived causality were dichotomized, i.e., all answers “Yes often” to symptom and of those, “Yes” answers to perceived causality to the office environment, were included in the analyses. Logistic regression analysis was also used to study associations of sex (women vs. men) and atopy (atopy vs. no atopy) on symptom reporting in years 2000–2020 compared to 1995–1999. The results are presented with odds ratios (OR) with 95% confidence intervals (CI). Considering the possibility that each respondent would not answer all questions in the questionnaire, an internal loss throughout the material was presupposed. The significance level was set at 0.05. Statistical analyses were performed with IBM SPSS Statistics version 27.

### 2.5. Ethical Considerations

This study was carried out in accordance with the Declaration of Helsinki. An application to the Swedish Ethical Review Authority was not necessary for this study since the material consisted of anonymous data, collected nationally in Sweden between 1995 and 2020. Questionnaire answers were collected and stored in a register with permission from the National Board of Health and Welfare, and the physical questionnaires destroyed. The respondents had not received information about the register. Answers are only shown at a group level, and since there is no code key they cannot be linked to a specific city, workplace, or person, and the risk of invasion of privacy is therefore low. This research can add information on symptoms in office environments and enable future research on the subject, for example, on groups with increased risk. Consequently, important knowledge can be added, concurrently, as the risk of harm to individuals is considered very low.

## 3. Results

### 3.1. Respondent Characteristics

General characteristics of the respondents and year groups are shown in Table 1. The five 5-year groups between 1995 and 2020 comprised 15.2%, 20.2%, 27.5%, 33.6%, and 3.0% of the total number of respondents (26,477), respectively. The sum of respondents was somewhat reduced due to internal loss between 0–5% per question in the questionnaire, with the highest loss found in questions regarding smoking habits. Women constituted an average of 62.8% of the respondents in the study, with an increased proportion for each year group, i.e., 52.4%, 59.0%, 62.3%, 69.4%, and 73.5%, respectively. Current or history of eczema, allergic rhinitis, and/or asthma was reported by 42.3% of the respondents. This proportion also increased throughout year groups, from 39.7% in 1995–1999 to 46.6% in 2015–2020. The proportion of smokers, on the contrary, decreased over the study period and was 6.9% in 2015–2020, i.e., about half of the proportion in 1995–1999 (15.7% smokers). The majority (58.7%) of respondents had worked at their current workplace for 1–5 years.

### 3.2. Reported Frequencies of Symptoms and Perceived Causality

Among all respondents, 50% reported having at least one of the 12 symptoms often (at least once every week), and 64.6% of those reported that they believed the work environment was the cause of the symptom. Among the 13,244 respondents from all year groups who reported at least one symptom, fatigue was the most frequently reported symptom during the time period, with an average of 58% of respondents, followed by heavy-feeling head (31.5%), itch/stinging/irritation in eyes (30.0%), irritated/congested/runny nose (28.2%), headache (22.2%), hoarseness/dry throat (19.7%), dry/red skin on face (19.7%), dry/itchy/red skin on hands (16.8%), scaly/itchy scalp or ears (14.6%), difficulties concentrating (12.4%), cough (11.6%), and nausea/dizziness (3.8%).

A perceived causality to the office environment among the reported symptoms was on average most often reported for itch/stinging/irritation in eyes (76.5%), followed by hoarseness/dry throat (72.4%), heavy-feeling head (71.2%), difficulties concentrating (69.4%), headache (64.0%), dry/red skin on face (61.2%), irritated/congested/runny nose (59.4%), cough (58.1%), fatigue (53.3%), dry/red skin on hands (46.6%), nausea/dizziness (45.0%), and scaly/itchy ears or scalp (35.4%). Year group frequencies and percentages of the 12 symptoms from the questionnaire and the perceived causality to the workplace for each symptom are presented in Table 2.

The reporting of general symptoms increased slightly over time, with the exception of nausea/dizziness (Table 2). The reporting of difficulties concentrating increased most and was approximately twice as high in the year group 2015–2020 compared to the year group 1995–1999. Of the mucous-membrane symptoms, the reporting of itchy, stinging, or irritated eyes and irritated, congested, or runny nose increased to some extent between 1995 and 2020. The symptoms hoarseness/dry throat and cough varied only slightly over the whole time period and were at about the same level in the year group 2015–2020 as in 1995–1999.

Of the three skin symptoms, the reporting of dry, itchy, or red skin on hands increased to some extent in the year group 2015–2020 compared to 1995–1999, whereas the other two symptoms varied slightly over the whole time period and were at about the same level in the year group 2015–2020 as in 1995–1999.

The reporting of the perceived causality of the symptoms to the office work environment decreased in general between 1995 and 2020. The only exception is the reporting of nausea/dizziness, which varied more over time and also increased in the year group 2015–2020 compared to 1995–1999.

### 3.3. Sex and Atopy

Logistic regression analyses were used to study the influence of sex and atopy on the reporting of symptoms (“Yes often”). Women had greater odds than men for reporting any of the 12 symptoms in the office environments of this study, as presented in Table 3. The effect of female sex showed in ORs ranging between 1.55 (scaly/itchy ears or scalp) and 3.06 (nausea or dizziness), all *p*-values < 0.001. Previous or current eczema, allergic rhinitis, or asthma—indicators of atopy—were also associated with greater odds for reporting any symptom in this study, with the smallest effect on difficulties concentrating, OR 1.49, and the greatest effect on scaly or itchy ears or scalp, OR 3.33, all *p*-values < 0.001 (Table 3). Sex had stronger associations with the first six symptoms compared to atopy, and conversely atopy was more strongly associated with the remaining six symptoms.

### 3.4. Logistic Regression Analyses Adjusted for Sex and Atopy

**Figure 1:**
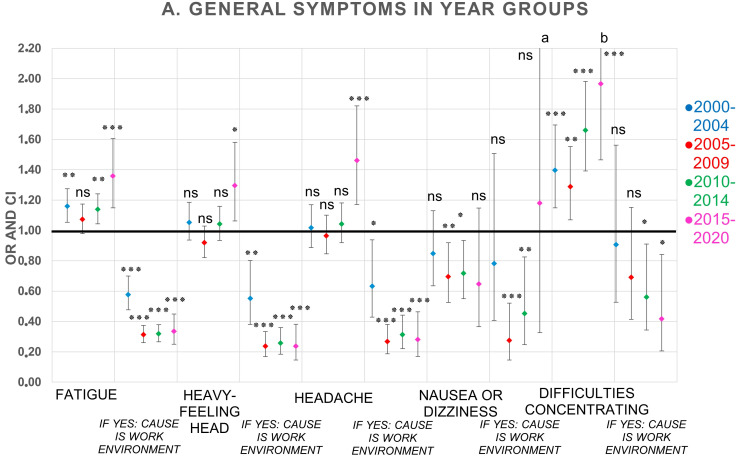
OR and 95% CI of 5-year groups compared to reference group (1995–1999, OR 1) for general symptoms and perceived causality to the office environment. Logistic regression analyses adjusted for sex and atopy. Number of respondents in each year group: 1995–1999 (*n* = 4028); 2000–2004 (*n* = 5348); 2005–2009 (*n* = 7293); 2010–2014 (*n* = 8904); 2015–2020 (*n* = 790). ^a^ 95% CI upper limit 4.27. ^b^ 95% CI upper limit 2.64. OR = odds ratio, CI = confidence interval. *p*-values: * <0.05; ** ≤0.01; *** ≤0.001; ns = not significant.

Associations between year groups and reported frequencies of symptoms and perceived causality to the office environment were examined with logistic regression analyses adjusted for sex and atopy, and with the earliest year group, 1995–1999, as reference (OR 1). Unadjusted analyses are shown in Supplementary Material (Table S1). Compared to the reference group 1995–1999, and as can be seen in Table 4 and Figure 1, the reporting of difficulties concentrating significantly increased in all year groups (ORs range between 1.29–1.97, *p* < 0.01), fatigue significantly increased in three of the year groups (ORs range between 1.14–1.36, *p* < 0.01), but not in 2005–2009 (*p* > 0.05), and heavy-feeling head (OR 1.30, *p* < 0.05), as well as headache (OR 1.46, *p* < 0.001), increased significantly only in the last year group, 2015–2020. The reporting of itchy, stinging, or irritated eyes significantly increased in the last year group (OR 1.47, *p* < 0.01) as well as irritated, congested, runny nose (OR 1.35, *p* < 0.01) and cough only in 2010–2014 (OR 1.26, *p* < 0.01). A significant decrease in reporting of hoarseness/dry throat occurred in three of the year groups between 2005 and 2020 (ORs range between 0.69–0.74, *p* < 0.05). Of the skin symptoms, only dry, itchy, or red skin on hands significantly increased in the last year group, 2015–2020 (OR 1.43, *p* < 0.01), having significantly decreased in year group 2005–2009 (OR 0.83, *p* < 0.05). Dry or red skin on face significantly decreased in 2005–2009 (OR 0.70, *p* < 0.001) and 2010–2014 (OR 0.76, *p* < 0.001).

**Figure 2:**
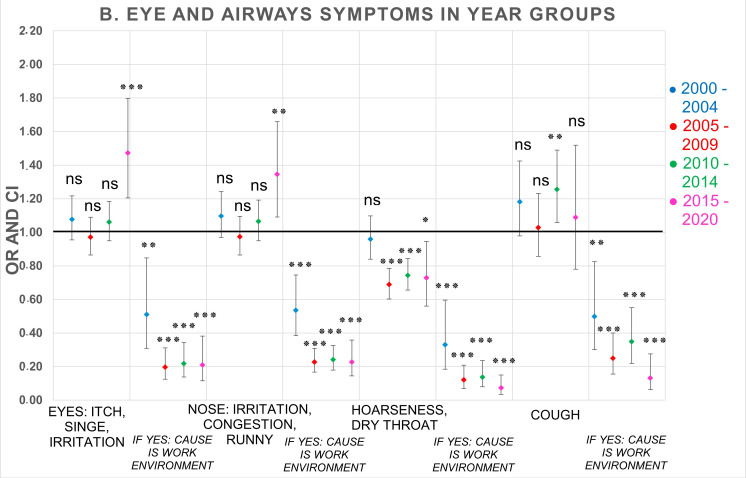
OR and 95% CI of 5-year groups compared to reference group (1995–1999, OR 1) for mucous-membrane symptoms and perceived causality to the office environment. Logistic regression analyses adjusted for sex and atopy. Number of respondents in each year group: 1995–1999 (*n* = 4028); 2000–2004 (*n* = 5348); 2005–2009 (*n* = 7293); 2010–2014 (*n* = 8904); 2015–2020 (*n* = 790). OR = odds ratio, CI = confidence interval. *p*-values: * <0.05; ** ≤0.01; *** ≤0.001; ns = not significant.

**Figure 3:**
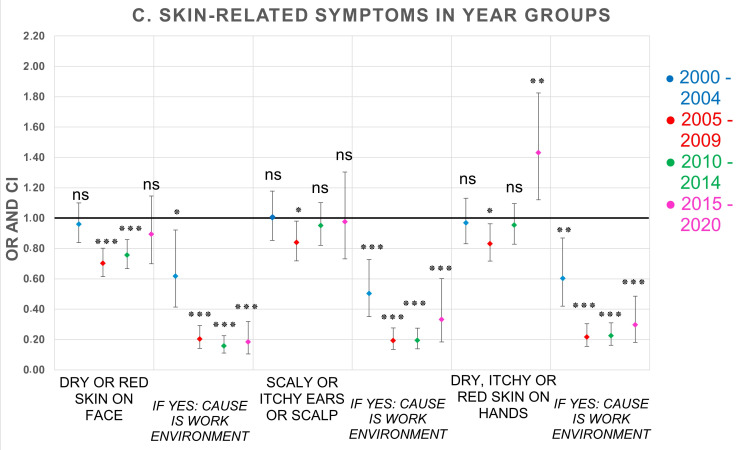
OR and 95% CI of 5-year groups compared to reference group (1995–1999, OR 1) for skin symptoms and perceived causality to the office environment. Logistic regression analyses adjusted for sex and atopy. Number of respondents in each year group: 1995–1999 (*n* = 4028); 2000–2004 (*n* = 5348); 2005–2009 (*n* = 7293); 2010–2014 (*n* = 8904); 2015–2020 (*n* = 790). OR = odds ratio, CI = confidence interval. *p*-values: * <0.05; ** ≤0.01; *** ≤0.001; ns = not significant.

In ten of 12 symptoms, the reporting of perceived causality to the office environment resulted in statistically significant decreased reporting in all year groups compared to the 1995–1999 year group (ORs range between 0.07–0.63, *p* < 0.05). Nausea/dizziness significantly decreased in 2005–2009 (OR 0.28, *p* < 0.001) and 2010–2014 (OR 0.45, *p* < 0.01), as did difficulties concentrating in 2010–2014 (OR 0.56, *p* < 0.05) and 2015–2020 (OR 0.42, *p* < 0.05). The significantly largest reductions over time (between 2005 and 2020) for reporting perceived causality to the office environment occurred for hoarseness/dry throat (ORs range between 0.07–0.14, *p* < 0.001), dry or red skin on face (ORs range between 0.16–0.20, *p* < 0.001), itchy, stinging, or irritated eyes (ORs range between 0.20–0.22, *p* < 0.001), irritated, congested, runny nose (ORs range between 0.23–0.24, *p* < 0.001), heavy-feeling head (ORs range between 0.24–0.26, *p* < 0.001), and cough (ORs range between 0.13–0.35, *p* < 0.001)—see Table 4 and Figure 1, Figure 2 and Figure 3. Further adjustment for number of years at the workplace together with sex and atopy was also performed (data not shown). Correlations differed slightly compared to adjustment for only sex and atopy, but there were no changes in statistical significance in any symptom. For that reason, the adjustment was not expanded further.

## 4. Discussion

The present study investigated changes in 12 reported symptoms and perceived causality of these symptoms to the office environment from MM 040 NA Office questionnaires completed between 1995 and 2020. In total, 50% of respondents in this study reported at least one symptom. In a Swedish study by Runeson-Broberg and Norbäck (2013), 18% of participants reported a work-related symptom [15]. In that study, answers were collected through postal questionnaires sent to a randomized population sample, where occupationally active respondents were included but types of occupation were not known. Furthermore, in an adult general population sample from Sweden (2015), 15% of people reported having at least one symptom from eyes, nose, airways, or general symptom at least once a week, which they attributed to the workplace or school [14]. The results from the previous studies are quite similar, and both are considerably lower than the 50% in the present study. In the present study, contact was initiated from the offices where the question of building-related symptoms had been raised, while in the previous studies workers were approached with a questionnaire. The different reasons and methods for contact and reporting symptoms probably explain the higher prevalence in the present study compared to the previous studies. On average, the most reported symptoms with a perceived causality to the office environment in the present study were itch/stinging/irritation in eyes, hoarseness/dry throat, heavy-feeling head, and difficulties concentrating, with a prevalence of about 70–76%. The least prevalent office-related symptoms were dry/red skin on hands, nausea/dizziness, and scaly/itchy ears or scalp, with a prevalence of about 35–46%. For comparison, eye irritation, respiratory, and general symptoms (i.e., primary headache) were also the most frequently reported symptoms (corresponding to a prevalence of 58.3, 45.3, and 47%, respectively) recently shown in the comprehensive OFFICEAIR project, covering 167 office buildings in eight European countries and comprising 1356 questionnaires [24].

The descriptive results show that the proportion of respondents with frequent symptoms at least once every week varies over time depending on the type of symptom. For the vast majority of symptoms, there are small changes in symptom prevalence between 1995 and 2014, but during the period 2015–2020, the prevalence increases somewhat more, mostly for difficulties concentrating, thereafter for dry, itchy, or red skin on hands, headache, itchy, stinging or irritated eyes, irritated, congested, or runny nose, heavy-feeling head, and fatigue. The results for the perceived causality of symptoms to the office environment show an opposite trend. For a majority of the symptoms, a decrease in reporting was seen over time—mostly for cough, hoarseness, or dry throat, dry or red skin on face, and irritated and congested or runny nose, but there are also reductions among the other symptoms.

Adjustments of data were performed for sex and atopy, both presumed to influence symptom reporting. There were more women than men in the present study, and women and persons with suggested atopy had greater odds for reporting symptoms in the office environments, comparing respondents of all years (2000–2020) to the reference year group (1995–1999). More symptoms reported among women are in accordance with other studies [7,12,14,15,25,26,27]. Furthermore, atopy was also a predictor of symptoms in previous studies where questionnaires were used to assess symptoms in workers [7,12,15]. As a consequence of the increased susceptibility to symptoms among atopics, it could plausibly be hypothesized that this group may serve as early indicators of symptom development at a group level. They might therefore be particularly important to study for this purpose.

Logistic regression analysis of time trends for reported symptoms adjusted for sex and atopy of the 12 examined symptoms in the present study showed some statistically significant increased reporting between 2000 and 2020 compared to the period 1995–1999. In particular, the results over time showed an increasing trend of difficulties in concentrating, but also fatigue. Furthermore, five of the symptoms—headache, heavy-feeling head, eyes, nose, and hands—increased significantly in the last year group 2015–2020 compared with 1995–1999. Conversely, hoarseness decreased significantly between 2005 and 2020, and nausea/dizziness between 2010 and 2014. The observed time trends strongly suggest a need for recurrent audit of the questionnaire, e.g., to enable capture of the most up-to-date reference data, in order to keep up with changes in symptom reporting in office environments over time. The outcome of the time trends was, however, not affected by the adjustments of data. Results from logistic regressions of unadjusted data are shown in Supplementary Material (Table S1). Therefore, it was concluded that the changes over time were influenced by factors other than sex and atopy.

Possible reasons for the observed time trends of reported symptoms in the present study could be multifactorial, involving, for example, workload of the office workers that may not have been consistent over time, as well as changes in office landscapes, construction, and building materials—affecting indoor climate and concentration patterns of air pollutants. Occupants of offices with higher concentrations of selected air pollutants have been shown to be more likely to report health symptoms in several recent studies [24,28,29,30], though no findings of such relationships have been reported [31]. Furthermore, trends in office layouts have changed during the last few decades, from cellular, private spaces, to more shared spaces and open-plan office landscapes [16]. Such changes in office layouts over time may have led to different perceived office environmental problems and could possibly have impacted time trends of symptoms in the present study. Previous studies have shown that workers in open-plan offices had more complaints regarding air quality and reported more symptoms, and office size was a predictor of symptoms [17,32]. Because of the anonymity of questionnaire answers from the database, it is not known what kind of office environments the respondents in the present study worked in.

Other possible reasons which might explain the time trends could stem from outside of the actual offices, especially considering the findings of this study that respondents in later year groups less often viewed the office environment as cause for their symptoms. Firstly, Internet usage and social media have become significantly more common in recent years, and many workers are often online or reachable for a large part of the day. Research on the effects of this on mental health, such as increased tiredness and depression, has increased in recent years [33]. This could explain increased symptom reporting and less attribution to the office in more recent year groups. Secondly, it is possible that attitudes or acceptance towards feeling unwell have changed during this time, which could explain increases of reported symptoms. In addition, changes over the time period in media reporting of indoor environment and building-related symptoms and illness could have impacted how the causes for symptoms are attributed. As seen in Figure 1, the biggest changes at year group level regarding perceived causality occur around the 2005–2009 group. It is possible that the popularity and interest in building-related symptoms, that had grown in the last few decades of the last century in terms of both research and media reporting, started to change around this time, which could affect how the office workers viewed their symptoms. The media’s impact on symptom reporting has been shown previously, in cases where the reporting of symptoms increased with extensive media reporting [34]. Thus, it is possible that different societal changes over time are reflected in the results of the present study. Further research is required to confirm the findings of the present study, to identify specific causes to the time trends, and enable suitable measures and preventive actions for improved health in office environments.

Limitations of the present study should also be discussed. The 2015–2020 year group included four more months than the other groups, but was the only group encompassing only four counties, resulting in a numerically smaller year group. Previous studies showed a slightly higher asthma prevalence in northern parts of Sweden (8.5% compared to 7%) [14], and another study found more skin-related symptoms in northern Sweden [12]. Geographical differences could potentially have influenced symptom reporting in the 2015–2020 year group. However, the statistically significant results on time trends and symptom frequencies in later year groups do not appear to have been affected by the smaller size of that year group. Compared to nationwide Swedish coverage, the included counties do also include both cities and rural areas, both of which have been shown to be of importance to the reporting of building-related symptoms [35]. The 2015–2020 group encompassing these counties could accordingly still be representative for trends of symptom reporting.

The intention in the present study was to include childhood and current eczema, rhinitis, and asthma, as this would suggest atopy. Atopy and the pathogenesis of development of asthma are linked. There are, however, non-allergic forms of eczema, rhinitis, and asthma, e.g., adult-onset asthma, which are less associated with atopy. In this study, answering “yes” to, e.g., asthma would automatically include the respondent in the “atopy group”, without distinguishing between types of asthma. Non-allergic asthma in one study was prevalent in 39% of adult asthmatics, and was more common among women than men [26]. Adult-onset asthma in previous studies was also more common among women [36,37]. However, persons with adult-onset asthma could pathophysiologically still have an increased sensitivity to office environmental exposures, and could consequently (and similarly to atopics) be prone to reporting symptoms earlier in these environments, and thus be just as fitted to follow trends over time. It could therefore be argued that differentiating types of asthma would not affect the results.

Self-administered questionnaires are based on subjectively based information, and exaggerations, understatements, and recall bias are all possible. Furthermore, internal loss is difficult to avoid with this sort of study design. However, the internal loss of 0–5.0% per question in this study with 26,477 questionnaires was considered an acceptable amount, with no significant impact on the results.

## 5. Conclusions

This study found changes over time where reporting of the majority of symptoms in the questionnaire increased in the later years. Conversely, the perceived causality to the office environment had decreased over time for all symptoms but one. The reasons behind the time trends are most likely multifactorial, involving the inherent reporting bias of self-administered questionnaire data and altered characteristics of the work environments, as well as societal changes related to the digital media landscape affecting health and the public’s view on causality with indoor environments. Further research is required to confirm these results, identify specific causes to the time trends, and enable suitable measures and preventive actions for improved health in office environments. Accordingly, the results strongly suggest a need for recurrent audit and development of the utilized questionnaire, e.g., enabling capture of the most up-to-date reference data, in order to keep up with changes in symptom reporting in office environments over time.

## Figures and Tables

**Table 1:** General characteristics and statistics of questionnaire respondents divided into year groups. The sum of respondents is less than 26,477 and differs within year groups because of internal loss in the questionnaire.

		Year Group				
Respondents		1995–1999 N = 4028	2000–2004 N = 5348	2005–2009 N = 7293	2010–2014 N = 8904	2015–2020 N = 790
Sex (%)	Men	1917 (47.6)	2194 (41.0)	2751 (37.7)	2725 (30.6)	209 (26.5)
	Women	2111 (52.4)	3154 (59.0)	4542 (62.3)	6179 (69.4)	581 (73.5)
Atopy: current or ever eczema, hay fever or asthma (%)	Yes No	1580 (39.7) 2399 (60.3)	2161 (41.1) 3091 (58.9)	2990 (41.9) 4143 (58.1)	3836 (44.0) 4890 (56.0)	362 (46.6) 414 (53.4
Current smoker (%)	Yes	615 (15.7)	726 (14.0)	752 (10.7)	718 (8.4)	52 (6.9)
	No	3302 (84.3)	4478 (86.0)	6282 (89.3)	7866 (91.6)	702 (93.1)
Age (%)	<30	478 (11.9)	517 (9.6)	760 (10.4)	789 (8.8)	58 (7.3)
	30–39	940 (23.3)	1296 (24.2)	1616 (22.1)	1836 (20.5)	167 (21.0)
	40–49	1200 (29.8)	1452 (27.1)	1869 (25.5)	2570 (28.7)	206 (25.9)
	50–59	1171 (29.0)	1701 (31.7)	2194 (30.0)	2473 (27.6)	228 (28.7)
	60–70	244 (6.1)	398 (7.4)	885 (12.1)	1294 (14.4)	135 (17.0)
Min, max, mean, SD		19, 68, 44, 11	19, 67, 45, 11	19, 70, 46, 11	19, 70, 46, 11	21, 70, 47, 11
Years at current workplace (%)	<1	443 (11.3)	439 (8.6)	731 (12.4)	987 (11.5)	51 (6.7)
	1–5	1966 (50.0)	3346 (65.4)	3651 (62.2)	4835 (56.2)	455 (59.6)
	6–10	799 (20.3)	500 (9.8)	861 (14.7)	1330 (15.5)	114 (14.9)
	11–20	508 (12.9)	456 (8.9)	376 (6.4)	958 (11.1)	111 (14.5)
	>20	218 (5.5)	374 (7.3)	255 (4.3)	492 (5.7)	33 (4.3)
Min, max, mean, SD		<1, 46, 6, 7	<1, 42, 5, 7	<1, 45, 5, 7	<1, 56, 6, 7	<1, 53, 6, 7

Abbreviations: min; minimum, max; maximum, SD; standard deviation.

**Table 2:** Year group frequencies and percentages of 12 symptoms and the perceived causality to the workplace for each symptom.

Symptoms	Response Categories	Year Group				
		1995–1999	2000–2004	2005–2009	2010–2014	2015–2020
Fatigue	Yes often (%)	1023 (25.9)	1552 (29.7)	2076 (28.7)	2744 (30.9)	281 (35.7)
If yes often: workplace is cause	Yes (%)	697 (68.1)	913 (58.8)	1006 (48.5)	1334 (48.6)	141 (50.2)
Heavy-feeling head	Yes often (%)	575 (14.7)	832 (16.1)	1070 (14.9)	1533 (17.4)	167 (21.3)
If yes often: workplace is cause	Yes (%)	472 (82.1)	638 (76.7)	720 (67.3)	1029 (67.1)	114 (68.2)
Headache	Yes often (%)	396 (10.1)	564 (10.9)	773 (10.7)	1072 (12.2)	131 (16.8)
If yes often: workplace is cause	Yes (%)	295 (74.5)	392 (69.5)	457 (59.1)	656 (61.2)	79 (60.3)
Nausea/dizziness	Yes often (%)	89 (2.3)	108 (2.1)	126 (1.8)	171 (1.9)	15 (1.9)
If yes often: workplace is cause	Yes (%)	51 (57.3)	58 (53.7)	40 (31.7)	71 (41.5)	9 (60.0)
Difficulties concentrating	Yes often (%)	167 (4.3)	313 (6.1)	419 (5.9)	666 (7.6)	71 (9.1)
If yes often: workplace is cause	Yes (%)	123 (73.7)	231 (73.8)	293 (70.0)	444 (66.7)	45 (63.3)
Itchy, stinging, irritated eyes	Yes often (%)	535 (13.6)	787 (15.1)	1035 (14.4)	1444 (16.3)	170 (21.9)
If yes often: workplace is cause	Yes (%)	443 (82.8)	635 (80.7)	763 (73.7)	1073 (74.3)	124 (72.9)
Irritated, congested, runny nose	Yes often (%)	506 (12.9)	753 (14.5)	982 (13.6)	1347 (15.2)	148 (19.0)
If yes often: workplace is cause	Yes (%)	365 (72.1)	491 (65.2)	531 (54.1)	757 (56.2)	77 (52.1)
Hoarseness, dry throat	Yes often (%)	438 (11.2)	582 (11.2)	632 (8.8)	874 (9.9)	77 (9.8)
If yes often: workplace is cause	Yes (%)	363 (82.9)	458 (78.7)	422 (66.8)	599 (68.5)	43 (55.8)
Cough	Yes often (%)	189 (4.9)	308 (5.9)	394 (5.5)	601 (6.8)	49 (6.3)
If yes often: workplace is cause	Yes (%)	133 (70.4)	189 (61.4)	199 (50.5)	356 (59.2)	19 (38.8)
Dry/red skin on face	Yes often (%)	430 (11.0)	579 (11.2)	627 (8.7)	873 (9.9)	94 (12.0)
If yes often: workplace is cause	Yes (%)	332 (77.2)	406 (70.1)	359 (57.3)	446 (51.1)	51 (54.3)
Scaly/itchy scalp or ears	Yes often (%)	288 (7.4)	396 (7.7)	489 (6.8)	700 (7.9)	64 (8.3)
If yes often: workplace is cause	Yes (%)	148 (51.4)	171 (43.2)	139 (28.4)	200 (28.6)	27 (42.2)
Dry, itchy, or red skin on hands	Yes often (%)	318 (8.2)	436 (8.4)	550 (7.7)	810 (9.2)	105 (13.5)
If yes often: workplace is cause	Yes (%)	208 (65.4)	239 (54.8)	218 (39.6)	319 (39.4)	49 (46.7)

Notes: Possible answers to each symptom were “Yes often”, “Yes sometimes”, and “No”. For causality to office environment as cause for the symptom, answers “Yes” from those answering “Yes often” were included.

**Table 3:** Logistic regression analyses of the effect of sex, i.e., women vs. men, and atopy on reporting of symptoms (“Yes often”) in office environments.

Symptoms	Sex/Atopy	Odds Ratio (OR)	95% Confidence Interval	*p* Value
Fatigue	Women	2.04	1.92–2.16	<0.001
	Atopy	1.61	1.53–1.70	<0.001
Heavy-feeling head	Women	2.45	2.25–2.65	<0.001
	Atopy	1.70	1.59–1.82	<0.001
Headache	Women	2.80	2.54–3.10	<0.001
	Atopy	1.55	1.43–1.68	<0.001
Nausea or dizziness	Women	3.06	2.41–3.89	<0.001
	Atopy	1.62	1.35–1.94	<0.001
Difficulties concentrating	Women	1.67	1.48–1.87	<0.001
	Atopy	1.49	1.35–1.66	<0.001
Itch, stinging, or irritation in eyes	Women	2.54	2.34–2.76	<0.001
	Atopy	1.82	1.70–1.95	<0.001
Irritation, congestion, or runny nose	Women	1.80	1.66–1.96	<0.001
	Atopy	2.50	2.33–2.69	<0.001
Hoarseness/dry throat	Women	2.11	1.91–2.33	<0.001
	Atopy	2.25	2.07–2.45	<0.001
Cough	Women	1.68	1.49–1.89	<0.001
	Atopy	2.47	2.22–2.75	<0.001
Dry or red skin on face	Women	2.17	1.97–2.40	<0.001
	Atopy	2.45	2.25–2.67	<0.001
Scaly/itchy ears or scalp	Women	1.55	1.39–1.73	<0.001
	Atopy	3.33	3.00–3.68	<0.001
Dry, itchy, or red skin on hands	Women	2.42	2.16–2.70	<0.001
	Atopy	3.00	2.73–3.30	<0.001

Notes: logistic regression analyses of all years (2000–2020) compared to the reference year group (1995–1999).

**Table 4:** Logistic regression analyses of questionnaire answers regarding symptoms and perceived causality to the office environment, compared to the first-year group (1995–1999, OR 1), and adjusted for sex and atopy. Questionnaires were divided into five 5-year groups based on year of filling in. OR = odds ratio, CI = confidence interval. Bold = significant at *p* < 0.05.

	Year Group							
Symptom	2000–2004		2005–2009		2010–2014		2015–2020	
	OR	95% CI	OR	95% CI	OR	95% CI	OR	95% CI
Fatigue	**1.16**	1.05–1.28	1.07	0.98–1.17	**1.14**	1.04–1.24	**1.36**	1.15–1.61
Workplace is cause	**0.58**	0.48–0.70	**0.31**	0.26–0.37	**0.32**	0.27–0.38	**0.33**	0.25–0.45
Heavy–feeling head	1.05	0.94–1.19	0.92	0.82–1.03	1.04	0.93–1.16	**1.30**	1.06–1.58
Workplace is cause	**0.55**	0.38–0.80	**0.24**	0.17–0.33	**0.26**	0.18–0.36	**0.24**	0.15–0.38
Headache	1.02	0.89–1.17	0.96	0.85–1.10	1.04	0.92–1.18	**1.46**	1.17–1.82
Workplace is cause	**0.63**	0.43–0.94	**0.27**	0.19–0.38	**0.31**	0.22–0.44	**0.28**	0.17–0.46
Nausea/dizziness	0.85	0.64–1.13	**0.69**	0.53–0.92	**0.72**	0.55–0.93	0.65	0.37–1.15
Workplace is cause	0.78	0.41–1.51	**0.28**	0.15–0.52	**0.45**	0.25–0.83	1.18	0.33–4.27
Difficulties concentrating	**1.40**	1.15–1.70	**1.29**	1.07–1.55	**1.66**	1.39–1.98	**1.97**	1.47–2.64
Workplace is cause	0.91	0.53–1.56	0.69	0.41–1.15	**0.56**	0.34–0.91	**0.42**	0.21–0.84
Itchy, stinging, irritated eyes	1.08	0.95–1.22	0.97	0.86–1.09	1.06	0.95–1.18	**1.47**	1.21–1.80
Workplace is cause	**0.51**	0.31–0.85	**0.20**	0.12–0.31	**0.22**	0.14–0.34	**0.21**	0.12–0.38
Irritated, congested, runny nose	1.10	0.97–1.24	0.97	0.86–1.10	1.06	0.95–1.19	**1.35**	1.09–1.66
Workplace is cause	**0.54**	0.38–0.75	**0.23**	0.17–0.31	**0.24**	0.18–0.33	**0.23**	0.14–0.36
Hoarseness/dry throat	0.96	0.84–1.10	**0.69**	0.60–0.79	**0.74**	0.66–0.84	**0.73**	0.56–0.95
Workplace is cause	**0.33**	0.18–0.60	**0.12**	0.07–0.21	**0.14**	0.08–0.24	**0.07**	0.04–0.15
Cough	1.18	0.98–1.43	1.03	0.86–1.23	**1.26**	1.06–1.49	1.09	0.78–1.52
Workplace is cause	**0.50**	0.30–0.83	**0.25**	0.16–0.40	**0.35**	0.22–0.55	**0.13**	0.06–0.28
Dry/red skin on face	0.96	0.84–1.10	**0.70**	0.61–0.80	**0.76**	0.67–0.86	0.89	0.70–1.15
Workplace is cause	**0.62**	0.41–0.92	**0.20**	0.14–0.29	**0.16**	0.11–0.23	**0.18**	0.11–0.32
Scaly or itchy scalp or ears	1.00	0.85–1.18	0.84	0.72–0.98	0.95	0.82–1.10	0.98	0.73–1.30
Workplace is cause	**0.50**	0.35–0.73	**0.19**	0.13–0.28	**0.19**	0.14–0.27	**0.33**	0.18–0.60
Dry, itchy, or red skin on hands	0.97	0.83–1.13	**0.83**	0.72–0.96	0.95	0.83–1.10	**1.43**	1.12–1.83
Workplace is cause	**0.60**	0.42–0.87	**0.22**	0.15–0.30	**0.22**	0.16–0.31	**0.30**	0.18–0.49

## Data Availability

The data underlying this article are available in the article and in its online Supplementary Materials.

## Supplementary Materials

The following supporting information can be downloaded at: https://www.mdpi.com/article/10.3390/ijerph191811434/s1. Table S1: Unadjusted logistic regression analysis of symptom reporting and perceived causality to the office environment.
