# Supplementary material for: Changes in Reported Symptoms Attributed to Office Environments in Sweden between 1995 and 2020
Source: Int J Environ Res Public Health. 2022 Sep 11;19(18):11434. doi: 10.3390/ijerph191811434 (PMC9517370; doi:10.3390/ijerph191811434)
Supplement: Supplementary file 1 [file ijerph-19-11434-s001.zip › ijerph-1878691-supplementary.pdf]

Symptom		Year-group 1995-1999	2000-2004		2005-2009		2010-2014		2015-2020	
Fatigue	count (%)	1023 (25.9)	1552 (29.7)		2076 (28.7)		2744 (30.9)		281 (35.7)	
	OR 95%(CI)	1	1.21	1.10-1.32	1.15	1.06-1.26	1.28	1.18-1.40	1.59	1.35-1.87
If yes often:	count (%)	697 (68.1)	913 (58.8)		1006 (48.5)		1334 (48.6)		141 (50.2)	
cause is work environment	OR	1	0.58	0.48-0.71	0.32	0.27-0.39	0.33	0.28-0.40	0.35	0.26-0.46
Heavy-feeling head	count (%)	575 (14.7)	832 (16.1)		1070 (14.9)		1533 (17.4)		167 (21.3)	
	OR	1	1.11	0.99-1.25	1.02	0.91-1.14	1.22	1.10-1.36	1.58	1.30-1.91
If yes often:	count (%)	472 (82.1)	638 (76.7)		720 (67.3)		1029 (67.1)		114 (68.2)	
cause is work environment	OR	1	0.55	0.38-0.80	0.25	0.18-0.35	0.27	0.19-0.37	0.26	0.16-0.41
Headache	count (%)	396 (10.1)	564 (10.9)		773 (10.7)		1072 (12.2)		131 (16.8)	
	OR	1	1.09	0.95-1.25	1.07	0.94-1.22	1.23	1.09-1.39	1.79	1.45-2.22
If yes often:	count (%)	295 (74.5)	392 (69.5)		457 (59.1)		656 (61.2)		79 (60.3)	
cause is work environment	OR	1	0.62	0.42-0.91	0.27	0.19-0.39	0.32	0.22-0.44	0.30	0.18-0.48
Nausea/dizziness	count (%)	89 (2.3)	108 (2.1)		126 (1.8)		171 (1.9)		15 (1.9)	
	OR	1	0.91	0.69-1.21	0.76	0.58-1.01	0.85	0.65-1.10	0.84	0.48-1.46
If yes often:	count (%)	51 (57.3)	58 (53.7)		40 (31.7)		71 (41.5)		9 (60.0)	
cause is work environment	OR	1	0.73	0.38-1.38	0.25	0.14-0.48	0.40	0.22-0.72	0.81	0.25-2.69
Difficulties concentrating	count (%)	167 (4.3)	313 (6.1)		419 (5.9)		666 (7.6)		71 (9.1)	
	OR	1	1.44	1.18-1.74	1.38	1.15-1.66	1.82	1.53-2.16	2.23	1.67-2.97
If yes often:	count (%)	123 (73.7)	231 (73.8)		293 (70.0)		444 (66.7)		45 (63.3)	
cause is work environment	OR	1	0.88	0.51-1.52	0.67	0.40-1.11	0.56	0.34-0.90	0.44	0.22-0.89
Itch, singe, irritation in eyes	count (%)	535 (13.6)	787 (15.1)		1035 (14.4)		1444 (16.3)		170 (21.9)	
	OR	1	1.13	1.01-1.27	1.06	0.95-1.19	1.24	1.11-1.38	1.78	1.47-2.16
If yes often:	count (%)	443 (82.8)	635 (80.7)		763 (73.7)		1073 (74.3)		124 (72.9)	
cause is work environment	OR	1	0.52	0.31-0.85	0.21	0.13-0.32	0.23	0.15-0.36	0.23	0.13-0.41
Irritation, congestion or runny nose	count (%)	506 (12.9)	753 (14.5)		982 (13.6)		1347 (15.2)		148 (19.0)	
	OR	1	1.14	1.01-1.29	1.06	0.95-1.19	1.21	1.08-1.35	1.58	1.29-1.94
If yes often:	count (%)	365 (72.1)	491 (65.2)		531 (54.1)		757 (56.2)		77 (52.1)	
cause is work environment	OR	1	0.56	0.40-0.78	0.25	0.18-0.34	0.27	0.20-0.37	0.25	0.16-0.39
Hoarseness/dry throat	count (%)	438 (11.2)	582 (11.2)		632 (8.8)		874 (9.9)		77 (9.8)	
	OR	1	1.01	0.88-1.15	0.76	0.67-0.87	0.87	0.77-0.99	0.87	0.67-1.12
If yes often:	count (%)	363 (82.9)	458 (78.7)		422 (66.8)		599 (68.5)		43 (55.8)	
cause is work environment	OR	1	0.33	0.18-0.59	0.12	0.07-0.21	0.14	0.08-0.23	0.07	0.04-0.15
Cough	count (%)	189 (4.9)	308 (5.9)		394 (5.5)		601 (6.8)		49 (6.3)	
	OR	1	1.24	1.03-1.49	1.13	0.95-1.36	1.43	1.21-1.69	1.31	0.95-1.82
If yes often:	count (%)	133 (70.4)	189 (61.4)		199 (50.5)		356 (59.2)		19 (38.8)	
cause is work environment	OR	1	0.52	0.32-0.86	0.27	0.17-0.42	0.38	0.24-0.60	0.16	0.07-0.32
Dry/red skin on face	count (%)	430 (11.0)	579 (11.2)		627 (8.7)		873 (9.9)		94 (12.0)	
	OR	1	1.02	0.89-1.17	0.77	0.68-0.88	0.89	0.79-1.00	1.11	0.88-1.41
If yes often:	count (%)	332 (77.2)	406 (70.1)		359 (57.3)		446 (51.1)		51 (54.3)	
cause is work environment	OR	1	0.60	0.41-0.90	0.21	0.14-0.29	0.17	0.12-0.23	0.19	0.11-0.33
Scaly or itchy ears or scalp	count (%)	288 (7.4)	396 (7.7)		489 (6.8)		700 (7.9)		64 (8.3)	
	OR	1	1.04	0.89-1.22	0.92	0.79-1.06	1.08	0.94-1.24	1.13	0.85-1.50
If yes often:	count (%)	148 (51.4)	171 (43.2)		139 (28.4)		200 (28.6)		27 (42.2)	
cause is work environment	OR	1	0.51	0.36-0.73	0.21	0.15-0.30	0.21	0.15-0.29	0.36	0.20-0.64
Dry, itchy or red skin on hands	count (%)	318 (8.2)	436 (8.4)		550 (7.7)		810 (9.2)		105 (13.5)	
	OR	1	1.04	0.89-1.21	0.94	0.81-1.08	1.14	1.00-1.31	1.76	1.39-2.22
If yes often:	count (%)	208 (65.4)	239 (54.8)		218 (39.6)		319 (39.4)		49 (46.7)	
cause is work environment	OR	1	0.61	0.42-0.87	0.22	0.16-0.31	0.23	0.17-0.31	0.28	0.17-0.46
